# Farnesol Has an Anti-obesity Effect in High-Fat Diet-Induced Obese Mice and Induces the Development of Beige Adipocytes in Human Adipose Tissue Derived-Mesenchymal Stem Cells

**DOI:** 10.3389/fphar.2017.00654

**Published:** 2017-09-20

**Authors:** Hye-Lin Kim, Yunu Jung, Jinbong Park, Dong-Hyun Youn, JongWook Kang, Seona Lim, Beom Su Lee, Mi-Young Jeong, Seong-Kyu Choe, Raekil Park, Kwang Seok Ahn, Jae-Young Um

**Affiliations:** ^1^College of Korean Medicine, Basic Research Laboratory for Comorbidity Regulation, Graduate School, Kyung Hee University Seoul, South Korea; ^2^Department of Science in Korean Medicine, Graduate School, Kyung Hee University Seoul, South Korea; ^3^Department of Microbiology and Center for Metabolic Function Regulation, School of Medicine, Wonkwang University Iksan, South Korea; ^4^Department of Biomedical Science and Engineering, Gwangju Institute of Science and Technology Gwangju, South Korea

**Keywords:** obesity, farnesol, beige adipocytes, hAMSCs, AMPK, UCP1

## Abstract

Brown adipocytes dissipate energy as heat and hence have an important therapeutic capacity for obesity. Development of brown-like adipocytes (also called beige) is also another attractive target for obesity treatment. Here, we investigated the effect of farnesol, an isoprenoid, on adipogenesis in adipocytes and on the browning of white adipose tissue (WAT) as well as on the weight gain of high-fat diet (HFD)-induced obese mice. Farnesol inhibited adipogenesis and the related key regulators including peroxisome proliferator-activated receptor γ (PPARγ) and CCAAT/enhancer binding protein α through the up-regulation of AMP-activated protein kinase in 3T3-L1 murine adipocytes and human adipose tissue-derived mesenchymal stem cells (hAMSCs). Farnesol markedly increased the expression of uncoupling protein 1 and PPARγ coactivator 1 α in differentiated hAMSCs. In addition, farnesol limited the weight gain in HFD obese mice and induced the development of beige adipocytes in both inguinal and epididymal WAT. These results suggest that farnesol could be a potential therapeutic agent for obesity treatment.

## Introduction

Terpenoids are a large class of organic chemicals and found in many higher plants as well as in insects and fungi. Particularly, a high number of terpenoids are produced in green and flowering plants (Pichersky and Raguso, 2016). The fundamental function of plant terpenoids is defense against biological enemies, and they have diverse roles as phytohormones, protein modification reagents, anti-oxidants (Kessler and Heil, 2011), and anti-inflammatory agents (Morikawa et al., 2017). Therefore, terpenoids, which include carotenoids, isoprenoids, and its alcohol isoprenols (polyprenyl alcohols), are contained in many plants for both herbal and dietary use (He et al., 1997; Park and Jang, 2017).

Farnesol, a typical isoprenol, has been reported to exhibit protective effects against many human cancers, such as lung, prostate, and colon cancer (Au-Yeung et al., 2008; Jones et al., 2013; Gliszczyńska et al., 2016). Farnesol also has a critical role in the amelioration of inflammatory reactions by regulating major inflammation-related factors including proinflammatory cytokines, cyclooxygenase-2, and nitric oxide synthase (Inoue et al., 2000; Ku and Lin, 2015; Santhanasabapathy et al., 2015). Additionally, farnesol supplementation modulates lipid profiles by reversing the aberrated low-density lipoprotein/high-density lipoprotein (HDL-c) and HDL-c/total cholesterol ratios in asthmatic mice (Ku and Lin, 2015). This report suggests the possibility that farnesol may ameliorate obesity. Actually, it has been reported that farnesol decreases serum triglycerides and improves metabolic abnormalities by regulating peroxisome proliferator-activated receptor α (PPARα) (Duncan and Archer, 2008; Goto et al., 2011).

Obesity is a result of excess fat accumulation and causes diverse adverse effects on human health. Mammalian fat tissues consist mainly of adipocytes and have important roles in regulating systemic energy balance. Adipocytes are divided into two classes of adipocytes: white and brown. Whereas white adipocytes store energy in the form of triglycerides, brown adipocytes burn energy to produce heat in response to various stimuli such as cold or β3 adrenergic receptor (β3AR) agonists (Wang and Seale, 2016). Brown adipocytes have many mitochondria and carry out efficient thermogenesis through mitochondrial uncoupling protein (UCP; Cannon and Nedergaard, 2004). Activation of brown adipocytes contributes to whole body energy expenditure and therefore is an attractive drug target for obesity treatment. However, the small amount of brown adipose tissue (BAT) in human adults certainly limits their use as an anti-obesity effect utilizing the brown adipocyte activity.

Browning of white adipocytes is also another attractive target for obesity treatment. White adipocytes can be developed to brown-like adipocytes (also called “beige” or “brite”), which are characterized as UCP1-expressing and mitochondrial-rich adipocytes. Although much remains unclear of the development of beige adipocytes, it is certain that cold exposure and some other stimuli leads to appearance of UCP1 positive adipocytes which share a *Myf5*^-/-^ origin with white adipocytes in white adipose tissue (WAT) depots of mice (Peirce et al., 2014; Wang and Seale, 2016). Despite the controversy of the specific origin of beige adipocytes, several studies have reported the possibility of transdifferentiation between white and beige adipocytes (Bartelt and Heeren, 2014; Obregon, 2014; Rodríguez et al., 2015). Stimuli inducing beige adipocyte development include synthetic compounds such as PPARγ or β3AR agonist, and even natural compounds like berberine or capsaicin (Harms and Seale, 2013; Song et al., 2017).

Because we could assume the possibility that farnesol is a potential therapeutic chemical for obesity treatment based on previous studies, we investigated the anti-obesity activity of farnesol *in vitro* and *in vivo*. For the first time, we demonstrated that farnesol inhibits adipogenic differentiation of human adipose tissue-derived mesenchymal stem cells (hAMSCs) as well as 3T3-L1 cell line. In addition, we found that farnesol induces UCP1 expression in hAMSCs and in the WAT of high-fat diet (HFD)-induced obese mice.

## Materials and Methods

### Chemical Reagents and Antibodies

Farnesol was purchased from Sigma Chemicals Co. (St. Louis, MO, United States). Farnesol was dissolved in dimethyl sulfoxide (DMSO). 3-Isobutyl-1-methylxanthine (IBMX), dexamethasone (Dex), insulin, indomethacin, compound C (CC), and Oil-Red O powder were purchased from Sigma (St. Louis, MO, United States). Dulbecco’s Modified Eagle’s Medium (DMEM), penicillin/streptomycin/glutamine (P/S/G), bovine serum (BS), and fetal bovine serum (FBS) were purchased from Gibco BRL (Grand Island, NY, United States). Anti-CCAAT/enhancer binding protein α (C/EBPα), anti-UCP1, anti-peroxisome proliferator-activated receptor γ coactivator 1 α (PGC1α), and anti-glyceraldehyde-3-phosphate dehydrogenase (GAPDH) antibodies were purchased from Santa Cruz Biotechnology (Santa Cruz, CA, United States). Antibodies for PPARγ, AMP-activated protein kinase alpha (AMPKα), pAMPKα, acetyl-CoA carboxylase (ACC), pACC, adiponectin, and lipin1 were purchased from Cell Signaling Technology (Beverly, MA, United States).

### Cell Culture and Differentiation

Murine 3T3-L1 mouse fiembryo broblasts were (American Type Culture Collection, Rockville, MD, United States) cultured and differentiated as previously described (Kim et al., 2014, 2015; Jeong et al., 2015). Briefly, 3T3-L1 cells were grown in DMEM containing 10% BS and 100 units/ml of P/S/G solution at 37°C in 5% CO_2_ at 95% humidity until cells were fully confluent. After 2 days from full confluence (day 0), the cells were differentiated by a 48 h incubation in differentiation medium consisting of DMEM plus 10% FBS containing 0.5 mM IBMX, 1 μM Dex, and 1 μg/ml insulin (MDI). At day 2, the cells were cultured in DMEM plus 10% FBS supplemented with 1 μg/ml insulin and various concentrations of farnesol (0.5 and 2 μmol/l) for another 48 h followed by fresh DMEM culture medium containing 10% FBS and 1 μg/ml insulin.

Human adipose tissue-derived mesenchymal stem cells (Cell Engineering for Origin, Seoul, South Korea) were grown in DMEM plus 10% FBS with 100 units/ml of P/S/G solution at 37°C in 5% CO_2_ at 95% humidity until full confluence. Two days after full confluence (day 0), the cells were differentiated by incubation for a total of 6 days in DMEM containing 0.5 mmol/l IBMX, 1 μmol/l Dex, 1 μmol/l insulin, 100 μmol/l indomethacin (differentiation media, DM), and 10% FBS, which was changed to fresh DM on day 3 for an additional 3 days of incubation. From day 6, the hAMSCs were incubated in DMEM plus 10% FBS and 1 μg/ml insulin for 72 h. From days 9–15, the culture medium (consisting of DMEM with 10% FBS and 1 μmol/l insulin) was changed every 3 days. Various concentrations of farnesol (0.5 and 2 μmol/l) were supplemented in the culture medium of day 6.

### Cytotoxicity Measurements

The cells were seeded (2 × 10^4^ cells per well) on 96-well plates and treated with various concentrations (1–5 μmol/l) of farnesol for 48 h. Cell viability was measured using the cell proliferation MTS Kit from the Promega Corporation (Madison, WI, United States) as previously described (Kim et al., 2015).

### Oil-Red O Staining

Intracellularly accumulated triglyceride was measured using the Oil-Red O staining assay as in a previous study (Zhang L. et al., 2014).

### RNA Extraction and Real-Time PCR

RNA extraction and real-time PCR were performed with the GeneAllR RiboEx Total RNA Extraction Kit (GeneAll Biotechnology, Seoul, South Korea), Power cDNA Synthesis Kit (iNtRON Biotechnology, Seongnam, South Korea), SYBR Green Power Master Mix (Applied Biosystems, Foster City, CA, United States), and the Step One Real-Time PCR System (Applied Biosystems, Foster City, CA, United States) according to the manufacturers’ instructions as previously described (Kim et al., 2015). The primers used in this study are provided in **Table 1**.

**Table 1:** The primer sequences used for real-time RT-PCR.

Target gene	Primer sequences	GenBank Accession No.
*mPparg*	5′-TTTTCAAGGGTGCCAGTTTC-3′ (sense)	NM_001127330
	5′-TTATTCATCAGGGAGGCCAG-3′ (antisense)	
*mCebpa*	5′-GCCGAGATAAAGCCAAACAA-3′ (sense)	NM_001287514
	5′-CCTTGACCAAGGAGCTCTCA-3′ (antisense)	
*mFabp4*	5′-CGTAAATGGGGATTTGGTCA-3′ (sense)	NM_024406
	5′-TCGACTTTCCATCCCACTTC-3′ (antisense)	
*mAdipoq*	5′-AGACCTGGCCACTTTCTCCTCATT-3′ (sense)	NM_009605
	5′-AGAGGAACAGGAGAGCTTGCAACA-3′ (antisense)	
*mResistin*	5′-TTCCTTGTCCCTGAACTGCT-3′ (sense)	NM_001204959
	5′-AGCTCAAGACTGCTGTGCCT-3′ (antisense)	
	5′-CGCCAAAGAATAACCTGGAA-3′ (sense)	NM_001130412
	5′-TGAAGACTCGCTGTGAATGG-3′ (antisense)	
*mGapdh*	5′-AACTTTGGCATTGTGGAAGG-3′ (sense)	NM_001289726
	5′-GGATGCAGGGATGATGTTCT-3′ (antisense)	
*hPPARG*	5′-TGAATGTGAAGCCCATTGAA-3′ (sense)	NM_001330615
	5′-CTGCAGTAGCTGCACGTGTT-3′ (antisense)	
*hCEBPA*	5′-TGTATACCCCTGGTGGGAGA-3′ (sense)	NM_001285829
	5′-TCATAACTCCGGTCCCTCTG-3′ (antisense)	
*hFABP4*	5′-GCATGGCCAAACCTAACATGA-3′ (sense)	NM_001442
	5′-CCTGGCCCAGTATGAAGGAAA-3′ (antisense)	
*hADIPOQ*	5′-CCTAAGCCAGACATCGGTGA-3′ (sense)	NM_001177800
	5′-GTAAAGCGAATGGGCATGTT-3′ (antisense)	
*hGAPDH*	5′-CAA GGC TGT GGG CAA GGT-3′ (sense)	NM_001256799
	5′-GGA AGG CCA TGC CAG TGA-3′ (antisense)	

### Protein Extraction and Western Blot Analysis

Western blot analyses were performed as described previously (Choi et al., 2015; Kim et al., 2017). Briefly, harvested cells or homogenized tissues were lysed in ice-cold RIPA buffer. After the protein concentration determination, equal amounts of total cellular protein were resolved by 6–12% SDS polyacrylamide gel electrophoresis and then transferred to a polyvinylidene difluoride (PVDF) membrane. Western blot analyses were done with polyclonal rabbit antibodies against PPARγ, C/EBPα, ADIPONECTIN, LIPIN1, AMPKα, pAMPKα, ACC, pACC, and PGC1α, with polyclonal goat antibody against UCP1, and with polyclonal mouse antibody against GAPDH. Then, the blots were incubated with proper horseradish peroxidase (HRP)-conjugated secondary antibodies (Jackson Immuno Research, West Grove, PA, United States) for 1 h at RT.

### Animal Experiments

Male C57BL/6J mice (4-week old) were purchased from Daehan Biolink Co. (Eumsung, South Korea). Mice were maintained on a 12 h light/dark cycle in a pathogen-free animal facility and provided with diet and water *ad libitum* for 1 week prior to the experiments. For obesity induction, the mice were fed a HFD (Rodent Diet D12492; Research Diet, New Brunswick, NJ, United States) consisting of 60% fat in accordance with our previously published report (Jeong et al., 2015). The mice were fed a HFD for 4 weeks before administration of farnesol. After obesity induction, the mice were divided into two groups (*n* = 5) which were fed with a HFD and a HFD plus farnesol (5 mg/kg/day) for an additional 8 weeks. A group fed a normal diet (ND) for 12 weeks was used as the normal control. After the experiment, the mice were starved overnight and sacrificed under CO_2_ anesthesia, and the tissues were collected for further analyses.

### Ethics Statement

All procedures used in the animal experiments were performed according to a protocol approved by the Animal Care and Use Committee of the Institutional Review Board of the Kyung Hee University (confirmation number: KHUASP (SE)-13-012).

### Immunofluorescence Staining

To evaluate the UCP1 and PGC1α immunofluorescence, after treatment with or without farnesol, the hAMSCs were washed three times with PBS and fixed with 4% formaldehyde for 15 min at RT. They were permeabilized with PBS containing 0.25% Triton X-100 for 10 min. After that non-specific binding was blocked by incubation with 5% BSA in PBS for 30 min. Then, they were incubated with UCP1 and PGC1α antibodies in 5% BSA in PBS overnight at 4°C followed by incubation with fluorescent secondary antibody Alexa Fluor 488 and Alexa Fluor 546 for 1 h. Finally, they were incubated with DAPI for 3 min to stain the nuclei. After staining, all the samples were mounted in mounting medium. Images were acquired on a fluorescence microscope (Logos Biosystems, Anyang, South Korea).

### Hematoxylin and Eosin (H&E) Staining

After the HFD mice were sacrificed, the inguinal WAT (iWAT) and epididymal WAT (eWAT) samples were fixed for 24 h in 4% formaldehyde buffer and then dehydrated and embedded in paraffin following a standard procedure previously described (Kim et al., 2015). The WAT tissues were cut into 5 μm sections and stained with H&E. Microscopic examinations were done using a regular light microscope (Olympus Co., Tokyo, Japan).

### Statistical Analysis

All data, expressed as the mean ± SEM, were calculated by the one-tailed Student’s *t*-test or the one-way ANOVA using the software SPSS 12 for Windows (SPSS Inc., Chicago, IL, United States). A value of *p* < 0.05 was considered to indicate statistical significance.

## Results

### Farnesol Inhibits Adipogenesis Through AMPK and Induces UCP1 Expression in 3T3-L1 Cells

**FIGURE 1:**
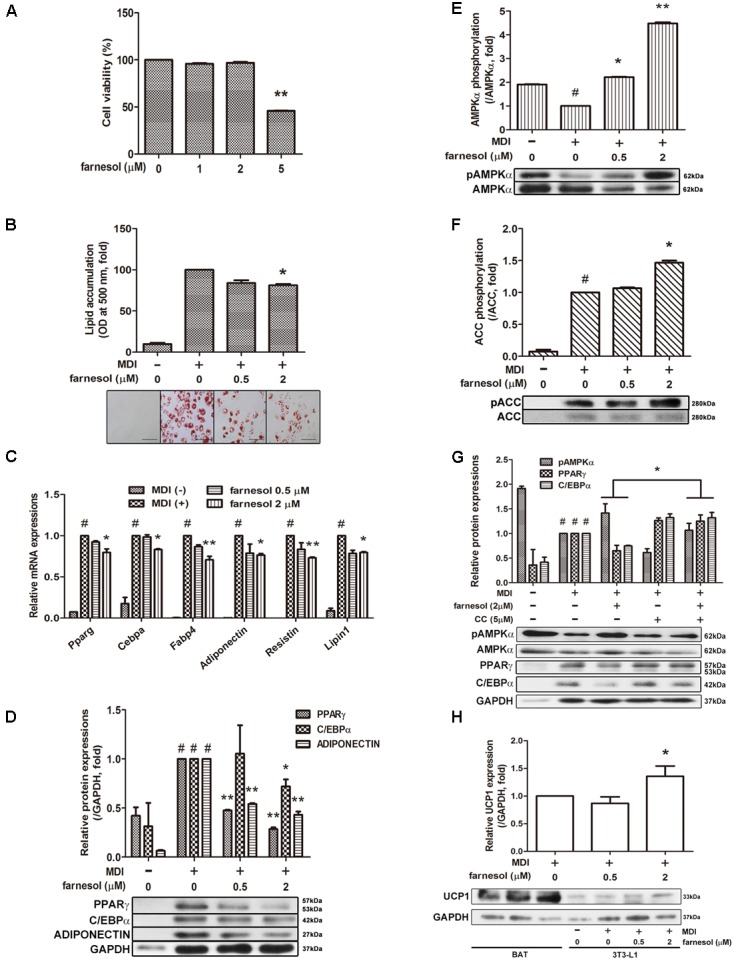
Farnesol inhibits white adipogenesis through AMPK in 3T3-L1 cells. **(A)** Cytotoxicity of farnesol in 3T3-L1 preadipocytes was determined by MTS assays. The cells were incubated without or with farnesol (1–5 μmol/l) for 48 h. Post-confluent 3T3-L1 cells were differentiated in the absence or presence of farnesol (0.5 and 2 μmol/l) for 6 days. **(B)** Intracellular lipid droplets were stained with Oil-Red O, observed at 200× (scale bar = 100 μm) and the absorbance was measured at 500 nm. **(C)** mRNA and **(D)** protein levels of adipogenesis-related factors in 3T3-L1 cells treated with farnesol. Phosphorylated levels of **(E)** AMPKα and **(F)** ACC in 3T3-L1 cells treated with farnesol. The pAMPKα and pACC protein expressions were normalized to the total AMPKα and ACC, respectively. **(G)** Compound C treatment diminished the farnesol-mediated AMPKα phosphorylation and PPARγ and C/EBPα inhibition. **(H)** Farnesol induced UCP1 expression in 3T3-L1 cells. All values are the means ± SEM of the data from three or more separate experiments, and statistical significance was calculated by the one-tailed Student’s *t*-test. ^#^*p* < 0.05, significantly different from MDI-uninduced preadipocytes; ^∗^*p* < 0.05 and ^∗∗^*p* < 0.01, significantly different from MDI-induced adipocytes.

To evaluate the cytotoxicity of farnesol, 3T3-L1 preadipocytes were treated with various concentrations of farnesol, and the cell viability was determined by an MTS assay. Treatment of 1 and 2 μmol/l of farnesol did not cause any cytotoxicity (**Figure 1A**). Based on this result, further farnesol treatments were done at concentrations of 0.5 and 2 μmol/l. Next, to investigate the anti-adipogenic effect of farnesol, the lipid accumulation was measured with the Oil-Red O staining assay. As shown in **Figure 1B**, 2 μmol/l of farnesol inhibited lipid accumulation in 3T3-L1 adipocytes (*p* < 0.05) suggesting that farnesol suppresses adipogenesis in 3T3-L1 cells. To investigate the anti-adipogenic mechanism of farnesol, its effects on the mRNA expression levels of *Pparg*, *Cebpa*, *Fabp4*, *Adipoq*, *Resistin*, and *Lipin1* were examined. The cells were exposed for 48 h to farnesol concentrations of 0.5 and 2 μmol/l. Farnesol (2 μmol/l) significantly suppressed the adipogenesis-related genes at both the mRNA and protein level (*p* < 0.05) (**Figures 1C,D**). Farnesol also activated the phosphorylation of AMPKα and ACC with statistical significance shown in **Figures 1E,F** (*p* < 0.05). To further confirm the involvement of AMPK in the anti-adipogenic effect of farnesol during the differentiation of 3T3-L1 adipocytes, we co-treated CC, an inhibitor of AMPK, with farnesol. As a result, the down-regulation effect of farnesol on the expressions of PPARγ and CEBPα disappeared by treatment of CC (**Figure 1G**). We also investigated whether farnesol could induce the browning of white adipocytes. Farnesol induced the expression of UCP1, a representative beige adipocyte-specific marker, at a concentration of 2 μmol/l in 3T3-L1 cells (*p* < 0.05) (**Figure 1H**).

### Farnesol Induces Beige Adipocyte-Specific Markers as well as Inhibits Adipogenic Factors in hAMSCs

**FIGURE 2:**
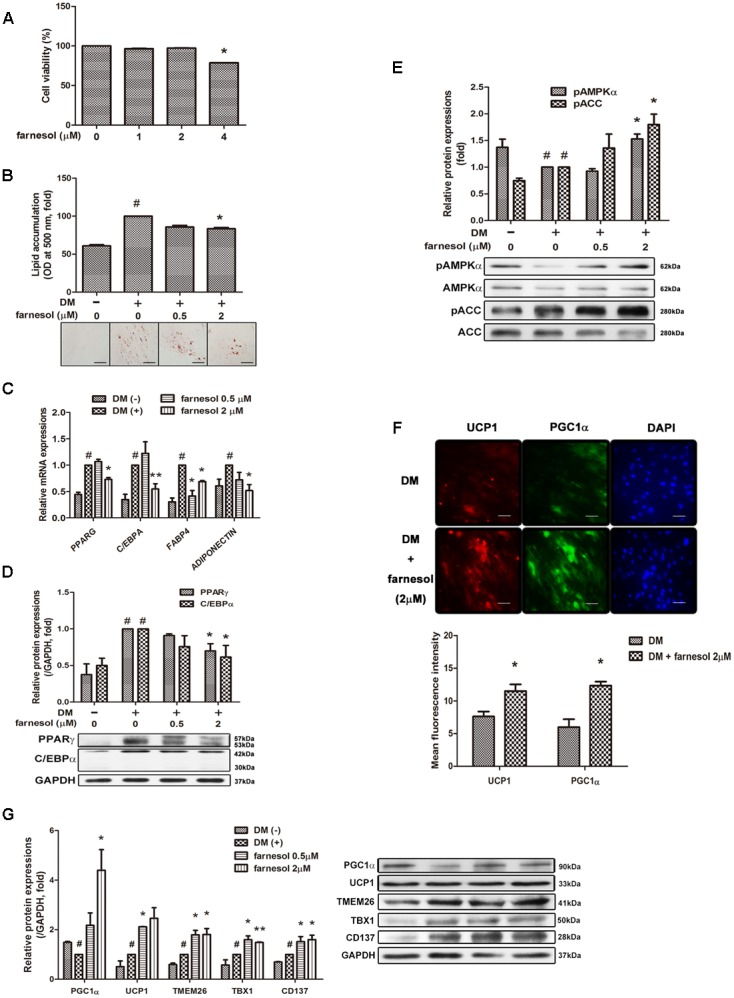
Farnesol induces development of beige adipocytes in human adipose tissue-derived mesenchymal stem cells (hAMSCs). **(A)** Cytotoxicity of farnesol in undifferentiated hAMSCs was determined by MTS assays. The cells were incubated without or with farnesol (1–4 μmol/l) for 48 h. Post-confluent hAMSCs were differentiated in the absence or presence of farnesol (0.5 and 2 μmol/l) for 15 days. **(B)** Intracellular lipid droplets were stained with Oil-Red O and the absorbance was measured at 500 nm. **(C)** mRNA and **(D)** protein levels of adipogenesis-related factors in hAMSCs treated with farnesol. **(E)** Phosphorylated levels of AMPKα and ACC in hAMSCs treated with farnesol. The pAMPKα and pACC protein expressions were normalized to the total AMPKα and ACC, respectively. **(F)** Representative images of immunostained UCP1 and PGC1α in hAMSCs treated with farnesol. The changes were observed at 200× (scale bar = 100 μm). **(G)** Protein levels of UCP1 and PGC1α in hAMSCs treated with farnesol. All values are the means ± SEM of the data from three or more separate experiments, and statistical significance was calculated by the one-tailed Student’s *t*-test. ^#^*p* < 0.05, significantly different from DM-uninduced preadipocytes; ^∗^*p* < 0.05, significantly different from DM-induced adipocytes.

Farnesol exhibited cytotoxicity at a 4 μmol/l concentration in hAMSCs (**Figure 2A**); thus, we selected a concentration of 0.5 and 2 μmol/l for the treatment of the hAMSCs. Farnesol also inhibited lipid accumulation in hAMSCs like in the 3T3-L1 cells (**Figure 2B**). The mRNA expression of the adipogenic factors *PPARG*, *CEBPA*, *FABP4*, and *ADIPOQ* was significantly inhibited by the treatment with farnesol at a concentration of 2 μmol/l (*p* < 0.05) (**Figure 2C**). The protein levels of PPARγ and C/EBPα were also confirmed in the hAMSCs by Western blot assay (**Figure 2D**). AMPK and ACC phosphorylations were increased significantly by treatment with farnesol while the total AMPK and ACC levels were unchanged shown in **Figure 2E** (*p* < 0.05). Consistently, as shown in the 3T3-L1 cells, farnesol promoted the progressive expressions of UCP1 and PGC1α during differentiation of the hAMSCs (*p* < 0.05) (**Figures 2F,G**).

### Farnesol Promotes UCP1 Expression in the WAT of HFD-Induced Obese Mice

**FIGURE 3:**
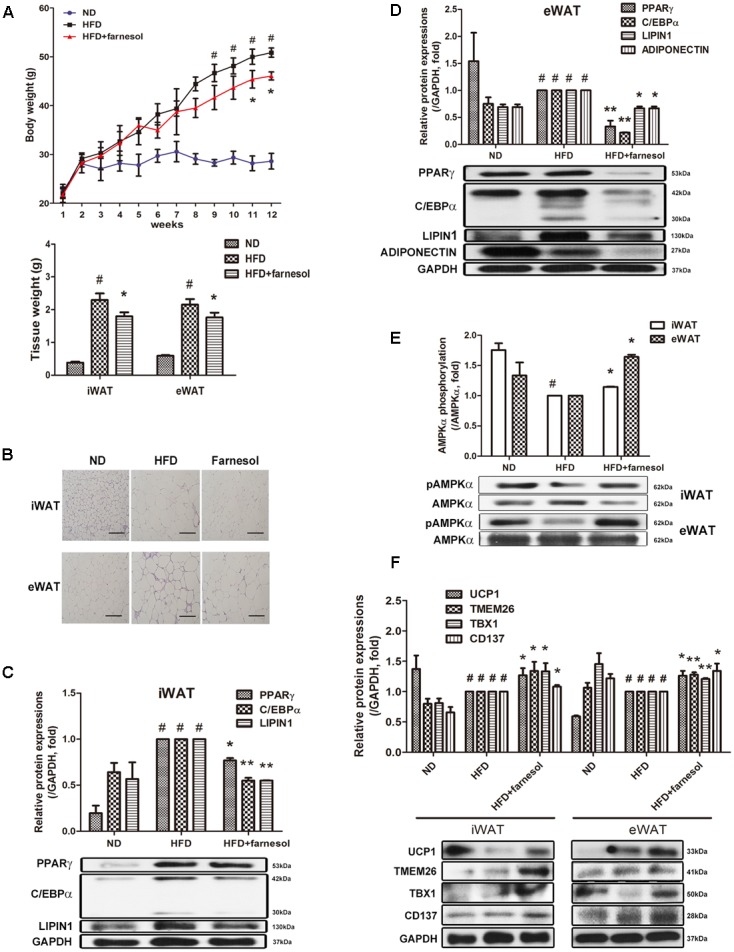
Farnesol induces browning of WAT in HFD-induced obese mice. **(A)** Body weight curve of the mice fed a ND (*n* = 5), HFD (*n* = 5), and HFD + farnesol (5 mg/kg/day) (*n* = 5) for 12 weeks. **(B)** H&E staining of the WAT sections from the mice after the termination of the study. The histological changes were observed at 200× (scale bar = 100 μm). mRNA and protein levels of adipogenesis-related factors in **(C)** inguinal WAT (iWAT) and **(D)** epididymal WAT (eWAT) from each group of mice. **(E)** Farnesol activates the phosphorylation of AMPKα in the iWAT and eWAT. The pAMPKα protein expression was normalized to the total AMPKα. **(F)** Farnesol induces UCP1 expression in the iWAT and eWAT. All values are the means ± SEM of the data from three or more separate experiments, and statistical significance was calculated by the one-way ANOVA **(A)** or one-tailed Student’s *t*-test **(C–F)**. ^#^*p* < 0.05, significantly different from the ND group; ^∗^*p* < 0.05 and ^∗∗^*p* < 0.01, significantly different from the HFD group. ND, normal diet-fed group; HFD, high-fat diet-fed group.

To investigate whether farnesol induces browning of WAT *in vivo* as well, C57BL/6 mice were fed farnesol and a HFD. Briefly, the mice were fed a HFD for 4 weeks to induce obesity before administration of farnesol. After induction of obesity, the mice were randomly divided into two groups: a HFD group with vehicle treatment and a HFD group with farnesol (5 mg/kg/day). The HFD plus farnesol group appeared to have significantly less weight gain than that of the HFD plus vehicle group (46.08 ± 0.42 vs. 50.86 ± 0.48 g, respectively). Additionally, the tissue weights and adipocyte sizes in both iWAT and eWAT were smaller than those of the HFD-induced obese mice (**Figures 3A,B**). Next, we investigated the expression of the adipogenesis-related factors PPARγ, C/EBPα, and LIPIN1 and the phosphorylated level of AMPK in both the iWAT and eWAT. Farnesol administration reduced the expressions of the factors and activated the phosphorylation of AMPK significantly (*p* < 0.05) (**Figures 3C–E**). Furthermore, farnesol significantly increased the expression of UCP1, the main factor of thermogenesis, and beige adipocyte-specific markers including TMEM26, TBX1, and CD137 in the iWAT and eWAT from HFD-induced obese C57BL/6 mice (*p* < 0.05) (**Figure 3F**).

## Discussion

Brown adipose tissue is the key site of non-shivering thermogenesis, and this thermogenic function is mainly mediated by UCP1. UCP1, which locates in the inner mitochondrial membrane of brown adipocytes, dissipates the proton gradients which are generated by the electron transport chain. This futile cycle decreases the mitochondrial membrane potential, which in turn elevates substrate oxidation of fatty acids, resulting in energy expenditure by generating heat (Cannon and Nedergaard, 2004; Kajimura et al., 2015).

Beige adipocytes can also express UCP1 and are found in the WAT depots of animals that have been exposed to cold and other inducers (Guerra et al., 1998; Harms and Seale, 2013). While brown adipocytes express high amounts of UCP1 even under nonstimulated conditions, beige adipocytes, when fully activated, can produce similar UCP1 levels as brown adipocytes by external and internal stimuli (Wu et al., 2012; Okamatsu-Ogura et al., 2013; Shabalina et al., 2013; Kajimura et al., 2015). Beige adipocytes express not only UCP1 but also brown adipocyte-specific genes such as *Cidea* and *Pgc1a* (encoding PGC1α) (Harms and Seale, 2013). These factors induce the expression of mitochondrial and fatty acid oxidation and thermogenic genes (Tiraby et al., 2003). In this study, farnesol induced UCP1 and PGC1α expression in human mesenchymal stem cells hAMSCs and in the WATs of HFD-induced obese mice (**Figures 2F,G**, **3F**). In addition, farnesol induced expressions of TMEM, TBX1, and CD137, beige-specific markers, in hAMSCs and in the WATs of HFD-induced obese mice (**Figures 2G**, **3F**). These results suggest that farnesol can induce beige adipocytes development through both directed differentiation from white adipocyte precursor cells and transdifferentiation from mature white adipocytes.

Moreover, farnesol treatment inhibited lipid accumulation and down-regulated adipogenesis-related genes such as *Pparg*, *Cebpa*, *Fabp4*, *Adiponectin*, *Resistin*, and *Lipin1* in 3T3-L1 cells and hAMSCs (**Figures 1C,D**, **2C**,**D**). The down-regulation of adipogenic factors by farnesol was confirmed in the iWAT and eWAT from the HFD-induced obese *in vivo* model (**Figures 3C,D**). In addition, body weight gain was decreased by farnesol administration to HFD-induced obese mice (**Figure 3A**). We used two cell culture model systems: fibroblast-like preadipocytes 3T3-L1 and hAMSCs. Farnesol induced the phosphorylation of AMPK in the two types of adipose cells and inhibited the adipogenic differentiation through the AMPK signaling pathway (**Figures 1G**, **2E**). Two major transcriptional modulators, PPARγ and C/EBPα, are involved in adipogenic differentiation in all types of adipose cells (Barak et al., 1999; Rosen et al., 1999). Moreover, the expression and activation of PPARγ and C/EBPα are regulated by the AMPK pathway indicating that the AMPK pathway has crucial roles during adipogenesis (Long and Zierath, 2006; Chen et al., 2011). Indeed, AMPK negatively regulates the expression of PPARγ and C/EBPα and inhibits preadipocyte differentiation and adipogenesis (Daval et al., 2006; Giri et al., 2006; Xu et al., 2017).

Our results show that phosphorylated AMPK was increased by farnesol administration in the iWAT and eWAT of the HFD-induced obese C57BL/6 mice (**Figure 3E**). In addition, in the iWAT and eWAT, expressions of UCP1 and several beige-specific markers such as TMEM26, TBX1, and CD137 expression were also increased by farnesol (**Figure 3F**). These results indicate that farnesol may drive the browning of WAT through AMPK activation. Our group reported that natural plant products prevent adipogenesis by activating AMPK *in vivo* and *in vitro* (Kim et al., 2014; Han et al., 2016; Lim et al., 2016). Other studies also have reported that AMPK is a target for berberine in the regulation of metabolism (Cheng et al., 2006; Lee et al., 2006). In addition, berberine drives browning of iWAT in *db/db* mice via AMPK activation (Zhang Z. et al., 2014). As far as the involvement of AMPK in the browning of WAT, however, distinct results have been reported. Vila-Bedmar et al. (2010) reported that the activation of AMPK with AICAR induced accumulation of UCP1-positive adipocytes in the eWAT of mice. Another study reported that treatment with AICAR did not increase the UCP1 expression within the WAT depots of male Wistar rats (Gaidhu et al., 2011). Therefore, further study is needed to elucidate whether farnesol induces brown adipocyte development of WAT through AMPK activation.

In conclusion, farnesol inhibited white adipogenesis through AMPK signaling and promoted beige adipocyte development in hAMSCs and the WAT of mice. Therefore, farnesol can exert multiple beneficial effects through the inhibition and browning of WAT.

## Author Contributions

H-LK, YJ, SL, and M-YJ performed the *in vitro* experiments. H-LK, M-YJ, JP, D-HY, BSL, and JK performed the mouse model experiment. S-KC, RP, and KSA provided technical and material support. H-LK collected the data. J-YU, JP, and H-LK wrote the manuscript. J-YU designed and supervised the study.

## Conflict of Interest Statement

The authors declare that the research was conducted in the absence of any commercial or financial relationships that could be construed as a potential conflict of interest.
